# Psychometrics of the Korean Version of the screen for adult anxiety related disorders (SCAARED)

**DOI:** 10.1186/s12888-024-05800-5

**Published:** 2024-05-30

**Authors:** Inae Hwang, Sanghoon Chin, Seongjun Mun, Min Ju You, Woori Moon, Silvia Kyungjin Lho

**Affiliations:** 1 Research and Development Division, 40FY Inc, Seoul, Republic of Korea; 2 Department of Psychiatry, McGill University, Montreal, QC Canada; 3 Department of Psychiatry, Seoul Metropolitan Government-Seoul National University Boramae Medical Center, Seoul, Republic of Korea

**Keywords:** Anxiety disorder, Scale, Screening, Psychometrics, Assessment, Korean

## Abstract

**Background:**

For enhanced management of anxiety disorders, early screening and accurate diagnostic differentiation are essential. The Screen for Adult Anxiety Related Disorders (SCAARED) has been developed to identify and categorize anxiety disorders, thereby facilitating timely and appropriate interventions. In line with this, we aimed to translate and validate the Korean version of the SCAARED questionnaire for the Korean population.

**Methods:**

The original SCAARED was translated into Korean and administered to community adult population (*N* = 119) ages 18–45 years old in South Korea. The internal consistency and test-retest reliability of the SCAARED were evaluated. In addition, its factor structure was examined using confirmatory and exploratory factor analysis. Concurrent validity was evaluated by comparing SCAARED with the Depression, Anxiety and Stress Scale-21 (DASS), the Beck’s Anxiety Inventory (BAI) and the State-Trait Anxiety Inventory (STAI). Test-retest reliability was evaluated one week after the first assessment.

**Results:**

The SCAARED showed good internal consistency (Cronbach’s α = 0.945) and test-retest reliability (γ = 0.883). The SCAARED had significant correlation with DASS-21 subscales (γ = 0.655–0.701), BAI (γ = 0.788) and STAI subscales (γ = 0.548–0.736), confirming good concurrent validity. The results of the Exploratory Factor Analysis showed four factors comparable to the original SCAARED (Generalized anxiety, Somatic/Panic/Agoraphobia, Social anxiety, and Separation anxiety). The area under the curve of the receiver operating characteristic of total and each of the factor scores ranged from 0.724 to 0.942.

**Conclusions:**

The Korean version of the SCAARED is a reliable and valid instrument to screen for anxiety disorders in the Korean adult populations.

**Supplementary Information:**

The online version contains supplementary material available at 10.1186/s12888-024-05800-5.

## Background

Anxiety disorders are among the most prevalent mental illnesses worldwide, affecting an estimated 4.05% of the global population, or roughly 301 million people as of 2019 [1]. Data from South Korea’s National Health Insurance System indicated that in 2021, approximately 3.1% of the nation’s total population was receiving treatment for anxiety disorders. Although 580,000 people are undergoing treatment in Korea, the actual number of people suffering from anxiety disorders is estimated to be approximately four to five times higher [2]. A systematic review estimated the economic burden of anxiety disorders to range from 0.25 to 0.78% of a country’s GDP [3]. This burden is potentially compounded by the increased likelihood that individuals with anxiety disorders have a heightened propensity to experience other mental health conditions, such as depression and substance use disorder [4, 5], suggesting a broader societal and economic impact. Given the substantial societal costs and undertreatment, there is a pressing need for efficient assessment tools that can easily screen and assess anxiety disorders in a clinical setting.

Several self-report assessment tools are currently used to evaluate anxiety, including the State-Trait Anxiety Inventory (STAI) [6], Beck Anxiety Inventory (BAI) [7], Depression, Anxiety, and Stress Scale-21 (DASS-21) [8], and Generalized Anxiety Disorder-7 (GAD-7) [9]. While these self-reporting assessments provide a quick measure of anxiety severity, they fall short of facilitating differential diagnosis among various anxiety disorders. Clinical assessments conducted by professionals include the HAM-A (Hamilton Anxiety Rating Scale) [10], but this mainly evaluates the level of anxiety phenomenologically. For detailed diagnostic categorization, a semi-structured interview such as the Structured Clinical Interview for DSM (SCID) [11–13] or the Mini-International Neuropsychiatric Interview (MINI) [14] is necessary. However, these assessments require trained professionals and take at least 20 to 40 min to administer.

To address these issues, the Screen for Adult Anxiety Related Disorders (SCAARED) was developed in alignment with DSM-5 anxiety disorder criteria [15]. This screening scale consists of a 44-item self-report scale adapted from the Screen for Children Anxiety Related Emotional Disorders (SCARED), which was initially designed for child and adolescent populations [16, 17]. Validation studies of SCARED have been conducted in various countries, and a meta-analysis examining its cross-cultural psychometric properties has further established the scale’s reliability and validity [18, 19]. The adult version, SCAARED, demonstrated a factor structure similar to that of SCARED, encompassing panic disorder/agoraphobia, generalized anxiety, social anxiety, and separation anxiety [15]. To date, aside from the original version, it has been validated in the Spanish, Persian, and Chinese populations [20–22].

In the current study, we evaluated the reliability and validity of the Korean version of the SCAARED within a Korean community population. We translated the SCAARED into Korean and conducted validation in a community sample, examining its internal consistency, test-retest reliability, concurrent validity, construct validity, and discriminant validity.

## Methods

### Participants

This study was conducted as part of a study of the Database for Emotion Analysis Using Physiological and Psychological Assessment by 40FY (DEAP-40FY), which is a comprehensive study examining the physiological, emotional, and psychological aspects of stress among Korean adults (https://clinicaltrials.gov/study/NCT06172673). We recruited adults between the ages of 19 and 45 who can read fluently in Korean. The sample comprised 119 community-dwelling participants (57.1% women, mean age = 32.2 ± 6.3; education = 15.9 ± 1.4 years, all Korean), recruited via advertisement among online communities in South Korea from July 19, 2023, to December 5, 2023 (Table 1).

**Table 1:** Demographic characteristics of the participants

	Mean	SD
Age	32.2	6.3
Gender		
Male	51 (42.9%)	
Female	68 (57.1%)	
Education (years)	15.9	1.4
Marriage		
Single	99 (83.2%)	
Married	20 (16.8%)	
Occupation		
Employed	60 (50.4%)	
Unemployed	31 (26.0%)	
Student	12 (10.1%)	
Housewife	4 (3.4%)	
Self-employed	12 (10.1%)	

SD, Standard Deviation

### Measures

The psychiatric symptoms were assessed using the Korean version of the SCAARED, BAI, DASS-21, and STAI-X.

The SCAARED is a 44-item self-report instrument that is used for screening DSM-5 anxiety disorders in adults. Each item is rated in the range of 0 to 2. With the consent of the author, the SCAARED [15] was translated into Korean by an experienced psychiatrist and a clinical psychologist. One translator was uninformed about the instrument’s concepts to ensure an unbiased approach. The translators reached a consensus on a forward translation, which was then back-translated by a bilingual individual, followed by necessary modifications. The final version was reviewed by the original translators.

The BAI [7] is a 21-item measure for evaluating anxiety severity on a 4-point Likert scale. A total score of 0–7 corresponds to normal anxiety, 8–15 to mild anxiety, 16–25 to moderate anxiety, and 26–62 to severe anxiety. The validity and reliability of the Korean version of the BAI have been demonstrated [23]. In the current study, the internal consistency was 0.91.

The DASS-21 [8] is the short form of the DASS-42, a self-report scale designed to measure negative emotions including depression, anxiety and stress. Each item is rated in the range of 0 to 3. The Korean version of the DASS-21 has been validated in clinical populations and among university students [24, 25]. In this study, the internal consistency was 0.93.

The STAI is a commonly used measure of trait and state anxiety [6] for diagnosing anxiety disorder. Form X comprises 20 items for assessing trait anxiety and another 20 for evaluating state anxiety severity, each rated on a 4-point Likert scale. Each item is rated in the range of 1 to 4. The validity and reliability of the Korean versions of STAI-X1 and X2 have been evaluated in both general and clinical populations [26–28]. In this study, the internal consistency was 0.75 for X1 and 0.81 for X2.

### Statistical analysis

IBM SPSS version 23 (IBM) was used for the statistical analyses. Initially, we performed descriptive statistical and psychometric analyses of the SCAARED items by calculating the means, standard deviation, range, kurtosis, symmetry, and corrected item-test correlation of all items on the scale. The internal consistency was calculated by Cronbach’s alpha coefficient. Test-retest reliability was calculated with a subsample of participants (*n* = 23) after one week. Subsequently, we conducted confirmatory factor analysis (CFA) to confirm the original model of the SCAARED [15]. Additionally, we performed exploratory factor analysis (EFA) to examine other possible factor structure of the SCAARED using principal axis factoring (PAF) with equimax rotation. The determination of the number of retained factors was based on the scree plot and parallel analysis [29]. To assess the adequacy of our data for factor analysis, we evaluated the Kaiser-Meyer-Olkin (KMO) measure of sampling adequacy and conducted Bartlett’s test of sphericity. Factor analysis was also completed using principal component analysis (PCA) with varimax rotation. A similar factor structure was found except for four items that were categorized into different factors (see Supplementary Table 1 in Additional file 1 for further details). Subsequently, concurrent validity was assessed using Pearson’s correlation of SCAARED scores and DASS-21 subscale scores, BAI total, and STAI-X1 and STAI-X2 scores. Last, discriminant validity was evaluated through receiver operating characteristic (ROC) analysis of the SCAARED total anxiety score and BAI score. The BAI was selected for its specificity in evaluating current anxiety symptoms over depression [7]. The diagnostic value was assessed by considering the criterion of reaching the cut-off point indicating moderate severity in the BAI (≥ 16).

## Results

### Reliability of the Korean version of SCAARED

#### Internal consistency and test-retest reliability

The descriptive statistics of the SCAARED items are presented in Table 2. The Korean version of the SCAARED and its back-translated version are included in Additional files 2 and 3, respectively. Except for three items, the corrected item-total correlations ranged between 0.30 and 0.70, which indicates an acceptable scale [30]. Internal consistency reliability was demonstrated for the total score and four subscale scores of the SCAARED. The Cronbach’s alpha coefficient for the SCAARED total score was 0.945. Those for the four subscales (Somatic/Panic/Agoraphobia, Generalized anxiety, Separation anxiety, Social anxiety) were 0.884, 0.926, 0.709, and 0.855, respectively. The test-retest correlations of total and subscales (Somatic/Panic/Agoraphobia, Generalized anxiety, Separation anxiety, Social anxiety) were 0.883 for the total score and 0.863, 0.875, 0.739, and 0.785 for each subscale, respectively, indicating the stability of the SCAARED scale.

**Table 2:** The Korean version of the SCAARED item scores and Cronbach’s alpha

Items	Response categories			Mean	SD	Range	Corrected item-total correlation	Alpha if item deleted
	0	1	2					
1	84	29	6	0.34	0.57	0–2	0.58	0.94
2	76	32	11	0.45	0.66	0–2	0.35	0.95
3	35	59	25	0.92	0.71	0–2	0.41	0.95
4	85	24	10	0.37	0.64	0–2	0.44	0.95
5	62	41	16	0.61	0.71	0–2	0.54	0.94
6	92	25	2	0.24	0.47	0–2	0.42	0.95
7	33	66	20	0.89	0.66	0–2	0.66	0.94
8	51	40	28	0.81	0.80	0–2	0.72	0.94
9	74	36	9	0.45	0.63	0–2	0.71	0.94
10	43	53	23	0.83	0.73	0–2	0.63	0.94
11	96	16	7	0.25	0.56	0–2	0.50	0.94
12	80	33	6	0.38	0.58	0–2	0.53	0.94
13	105	12	2	0.13	0.39	0–2	0.27	0.95
14	34	53	32	0.98	0.75	0–2	0.65	0.94
15	81	34	4	0.35	0.55	0–2	0.45	0.94
16	95	22	2	0.22	0.45	0–2	0.36	0.95
17	93	19	7	0.28	0.57	0–2	0.59	0.94
18	38	66	15	0.81	0.64	0–2	0.53	0.94
19	82	35	2	0.33	0.51	0–2	0.45	0.94
20	84	24	11	0.39	0.65	0–2	0.50	0.94
21	31	59	29	0.98	0.71	0–2	0.64	0.94
22	75	31	13	0.48	0.69	0–2	0.43	0.95
23	48	42	29	0.84	0.79	0–2	0.71	0.94
24	48	44	27	0.82	0.78	0–2	0.54	0.94
25	88	27	4	0.29	0.53	0–2	0.57	0.94
26	106	11	2	0.13	0.38	0–2	0.20	0.95
27	77	28	14	0.47	0.70	0–2	0.47	0.94
28	82	32	5	0.35	0.56	0–2	0.60	0.94
29	63	31	25	0.68	0.80	0–2	0.66	0.94
30	84	30	5	0.34	0.56	0–2	0.17	0.95
31	61	39	19	0.65	0.74	0–2	0.65	0.94
32	92	22	5	0.27	0.53	0–2	0.41	0.95
33	67	47	5	0.48	0.58	0–2	0.33	0.95
34	42	55	22	0.83	0.72	0–2	0.44	0.95
35	29	65	25	0.97	0.68	0–2	0.61	0.94
36	94	20	5	0.25	0.52	0–2	0.39	0.95
37	29	55	35	1.05	0.74	0–2	0.77	0.94
38	99	17	3	0.19	0.46	0–2	0.54	0.94
39	48	57	14	0.71	0.67	0–2	0.60	0.94
40	90	26	3	0.27	0.50	0–2	0.41	0.95
41	25	55	36	1.07	0.73	0–2	0.47	0.94
42	41	59	19	0.82	0.69	0–2	0.52	0.94
43	40	58	21	0.84	0.70	0–2	0.49	0.94
44	45	48	26	0.84	0.76	0–2	0.57	0.94

SCAARED, Screen for Adult Anxiety Related Disorders; SCAARED severity scores: 0 = Not true or Hardly ever true, 1 = Somewhat true or sometimes true, 2 = Very true or often true

### Validity of the Korean version of SCAARED

#### Concurrent validity

Table 3 shows Pearson’s correlations between the SCAARED and the DASS-21, BAI, and STAI-X1 and STAI-X2. A Bonferroni-corrected *p*-value of < 0.002 denoted statistical significance. SCAARED total and sub-dimension scores showed significant positive correlations with DASS-21 depression, anxiety, and stress, BAI, and STAI-X1 and STAI-X2. However, the correlation between DASS-21 stress and SCAARED social, as well as between DASS-21 depression and SCAARED separation, did not reach the Bonferroni-corrected *p*-value.

**Table 3:** Correlations between SCAARED dimensions and DASS-21, BAI and STAI

	SCAARED									
	Total		Somatic/Panic/Agoraphobic		Generalized anxiety		Separation anxiety		Social anxiety	
	γ	*p*	γ	*p*	γ	*p*	γ	*p*	γ	*p*
DASS-21 depression	0.655	< 0.001	0.606	< 0.001	0.617	< 0.001	0.266	0.003	0.472	< 0.001
DASS-21 anxiety	0.701	< 0.001	0.725	< 0.001	0.600	< 0.001	0.422	< 0.001	0.419	< 0.001
DASS-21 stress	0.659	< 0.001	0.665	< 0.001	0.634	< 0.001	0.409	< 0.001	0.279	0.0022
BAI	0.788	< 0.001	0.809	< 0.001	0.704	< 0.001	0.446	< 0.001	0.440	< 0.001
STAI-X1	0.548	< 0.001	0.435	< 0.001	0.550	< 0.001	0.313	0.001	0.338	< 0.001
STAI-X2	0.736	< 0.001	0.594	< 0.001	0.749	< 0.001	0.344	< 0.001	0.531	< 0.001

SCAARED, The Screen for Adult Anxiety Related Disorders; DASS, The Depression Anxiety Stress Scale; BAI, The Beck Anxiety Inventory; STAI, The State-Trait Anxiety Inventory

### Construct validity

A confirmatory factor analysis with IBM SPSS Amos 26.0 was conducted to assess the fit of the original model of the SCAARED [15]. The model fit indices were as follows: Chi-square minimum (CMIN) was 1396.04 with degrees of freedom (df) = 884 (*p* < 0.01). The ratio of chi-square minimum to degrees of freedom (CMIN/df) was 1.58. Additional fit indices included the standardized root mean square residual (SRMR) at 0.079, the goodness of fit index (GFI) at 0.69, the comparative fit index (CFI) at 0.80, and the root mean square error of approximation (RMSEA) at 0.070. These results indicated inadequate fit of the data to the original SCAARED model. As a result, we performed an exploratory factor analysis using PAF with equimax rotation. Eleven factors had initial eigenvalues greater than 1, ranging from 1.046 to 13.60. The scree plot showed a sharp point inflection after fifth factor. Lastly, the parallel analysis indicated the number of appropriate factors to be retained was four, which was consistent with the original model. We forced a solution with 4 factors. The analyses yielded four clinically interpretable factors that explained 42.6% of the variance. The KMO measure of sampling adequacy was 0.823, and Bartlett’s test of sphericity was significant (χ2 = 3054, df = 946, *p <* 0.001). The final factor analysis result with PAF method is shown in Table 4. The first factor replicates the construct of Generalized anxiety including 14 items (5, 7, 8, 9, 14, 21, 23, 24, 29, 31, 35, 37, 39 and 44); the second factor rebuilds the construct of Social anxiety including 8 items (3, 10, 27, 34, 38, 41, 42 and 43). The third factor includes items related to Somatic/Panic/Agoraphobia, composed of 13 items (1, 2, 6, 11, 12, 15, 17, 22, 25, 28, 32, 36 and 40) and the fourth factor is defined by the items of Separation anxiety, composed of 9 items (4, 13, 16, 18, 19, 20, 26, 30 and 33).

**Table 4:** Factor analysis for the four-factor solution of the Korean version of the SCAARED (PAF method)

Item	Factor I Generalized anxiety	Factor II Social anxiety	Factor III Somatic/Panic/ Agoraphobia	Factor IV Separation anxiety
08. It is hard for me to stop worrying	0.745			
23. I am a worrier	0.701			
21. I worry about things working out for me	0.667			
39. I worry about things that have already happened	0.635			
31. When I worry a lot, I feel restless	0.613			
07. I am nervous	0.598			
37. I worry about how well I do things	0.598	0.350	0.362	
29. People tell me that I worry too much	0.577			
14. I worry about being as good as other people	0.551			
35. I worry about what is going to happen in the future	0.549			
09. People tell me that I look nervous	0.508	0.363		
05. I worry about people liking me	0.504	0.378		
24. When I worry a lot, I have trouble sleeping	0.463			
44. When I worry a lot, I feel irritable.	0.431		0.363	
42. I feel nervous when I go to parties, dances, or any place where there will be people that I don’t know well		0.778		
27. It is hard for me to talk with people I don’t know well		0.762		
10. I feel nervous with people I don’t know well		0.718		
34. I feel shy with people I don’t know well		0.646		
03. I don’t like to be with people I don’t know well		0.581		
43. I am shy		0.556		
38. I am afraid to go outside or to crowded places by myself		0.452	0.369	
41. I feel nervous when I am with other people and I have to do something while they watch me (for example: speak, play a sport)	0.364	0.418		
06. When I get anxious, I feel like passing out			0.629	
40. When I get anxious, I feel dizzy			0.624	
11. I get stomachaches at school, at work, or in public places			0.541	
15. When I get anxious, I feel like things are not real			0.538	
01. When I feel nervous, It is hard for me to breathe			0.515	
25. I get really frightened for no reason at all	0.306		0.492	
36. When I get anxious, I feel like throwing up			0.478	
02. I get headaches when I am at school, at work or in public places			0.451	
28. When I get anxious, I feel like I’m choking			0.44	
12. When I get anxious, I feel like I’m going crazy	0.415		0.434	
17. I worry about going to work or school, or to public places		0.338	0.415	0.328
32. I am afraid of having anxiety (or panic) attacks			0.382	0.305
22. When I get anxious, I sweat a lot		0.318	0.365	
33. I worry that something bad might happen to my family				0.549
16. I have nightmares about something bad happening to my family				0.54
04. I get nervous if I sleep away from home				0.516
13. I worry about sleeping alone				0.513
30. I don’t like to be away from my family				0.463
26. I am afraid to be alone in the house				0.429
18. When I get anxious, my heart beats fast				0.388
20. I have nightmares about something bad happening to me	0.308			0.375
19. I get shaky				0.322
Eigenvalue	13.1	2.34	1.89	1.45
% of variance	29.7	5.36	4.29	3.29

PAF, principal axis factoring; SCAARED, Screen for Adult Anxiety Related Disorders; All loading greater than 0.30 are reported

Factor I (Generalized anxiety): Items 5, 7, 8, 9, 14, 21, 23, 24, 29, 31, 35, 37, 39, 44

Factor II (Social anxiety): Items 3, 10, 27, 34, 38, 41, 42, 43

Factor III (Somatic/Panic/Agoraphobia): Items 1, 2, 6, 11, 12, 15, 17, 22, 25, 28, 32, 36, 40

Factor IV (Separation anxiety): Items 4, 13, 16, 18, 19, 20, 26, 30, 33

### Discriminant validity

**Fig. 1:**
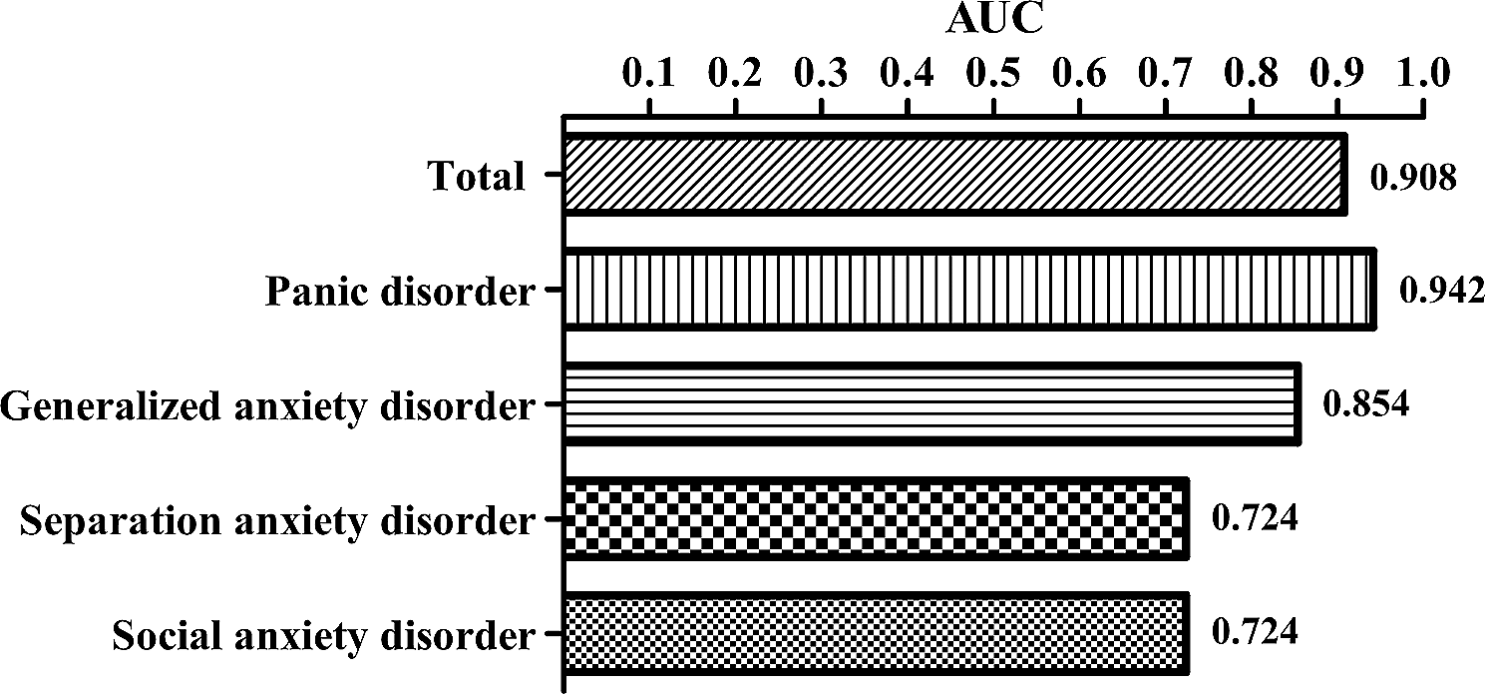
Receiver Operating Characteristic (ROC) Analysis. AUC, Area Under the Curve

The diagnostic value was assessed by taking as a criterion having reached the cut-off point of moderate severity in the BAI. The ROC curve was examined for each of the SCAARED subscales and for the total score. The area under the curve (AUC) for the total anxiety were 0.908, which could be considered very accurate. Those for GAD, Panic/Somatic, Social anxiety, and Separation anxiety were 0.854, 0.942, 0.724, and 0.724, respectively (all *p*-values < 0.001) (Fig. 1).

## Discussion

The aim of this study was to examine the reliability and validity of the SCAARED in a Korean community population. We showed that the internal consistency, test-retest reliability, and concurrent validity of the instrument were good. Additionally, the construct validity was proved by PAF analysis, which confirmed that the instrument was valid. Our results demonstrated good discriminant validity.

The internal consistency examined by the Cronbach’s alpha coefficients for the SCAARED total score, Somatic/Panic/Agoraphobia, Generalized anxiety, Separation anxiety, and Social anxiety score were excellent. The Cronbach’s alpha coefficients for the SCAARED total score reported in the validation study of the original, Spanish, Chinese, and Persian version of the SCAARED were 0.97, 0.91, 0.94, and 0.97, respectively, indicating that our result was comparable to these studies [15, 20–22]. The concurrent validity was shown by a significant correlation with the DASS-21, BAI, and STAI, which was comparable to the validation study of the Spanish version [20].

Construct validity of our study was assessed through factor analysis, identifying four factors (Somatic/Panic/Agoraphobia, General anxiety, Separation anxiety, Social anxiety) in the SCAARED, aligning with the dimensional structure of both the original and other versions of SCAARED [15, 20–22], corresponding to the four factors of the Screen for Child Anxiety Related Emotional Disorders (SCARED) [16]. However, 4 items (9, 18, 19, and 38) were assigned to different factors compared to the original SCAARED. Item 38, initially in the Somatic/Panic/Agoraphobia factor, moved to Social anxiety, also showing cross-loading on Separation anxiety. This reassignment is justified as the item addresses exposure to social situations and separation from loved ones. This trend was consistent with Spanish and Chinese validation studies, where item 38 loaded highest on Social anxiety [20, 22]. Items 9, originally under Somatic/Panic/Agoraphobia, showed the highest loading on Generalized anxiety, possibly due to the inherent overlap between anxiety disorders. Items 18 and 19, initially in the Panic Disorder factor, were reassigned to Separation anxiety, aligning with themes of high anxiety.

The discriminant validity of the Korean version of SCAARED, assessed using AUC values from the ROC curve, was found to be satisfactory. These results imply that the Korean version of SCAARED demonstrates a performance analogous to other versions of the SCAARED scales, including the original and Spanish versions [15, 20], suggesting its utility for screening anxiety disorders in the Korean adult population.

Other anxiety scales reflect the overall severity of anxiety, encompassing physical symptoms, cognitive symptoms, and associated depression [6–8, 10], making it challenging to obtain diagnostic information. Moreover, scales such as the Panic Disorder Severity Scale [31, 32], utilized in Korea, are designed to reflect the severity of specific disorders like panic disorder. Consequently, to categorize diagnoses, clinical assessments such as the SCID or MINI are delivered by trained clinicians [11, 14]. The SCAARED, on the other hand, offers diagnostic classification information and allows for the administration of survey assessments within a relatively brief period, presenting a significant advantage in clinical settings. Therefore, the usefulness of SCAARED includes assisting clinicians by providing an initial diagnostic impression, allowing them to focus more on additional questions necessary for diagnosing anxiety disorders. Furthermore, by providing community members and individuals seeking help with a diagnostic impression of which anxiety disorder they are likely experiencing, it enhances disease awareness and facilitates the process of seeking psychiatric treatment.

This study has several limitations. First, our study was based on a relatively small sample size, which might have resulted in an inadequate fit of the CFA, and thus we could not confirm the original factor structure suggested in the original version of the SCAARED [15]. Despite this, the congruence of factor structures observed in cross-cultural validations of the SCAARED [15, 20–22] provides provisional support for our findings. Nevertheless, future studies employing larger sample sizes are crucial for more definitive validation of the factor structures of the Korean version of the SCAARED. Second, since we based our study on a community sample interested in mental health services, concerns about generalizability arise. Participants were recruited as part of a study focused on mental health services addressing stress and anxiety. It is highly likely that individuals with a strong interest in mental health, especially those experiencing emotional depression or anxiety, were chosen as participants. Therefore, it can be understood that we recruited a community sample requiring mental health services rather than a general population. This is reflected in the fact that the mean scores for BAI, STAI, and DASS-21 are at a mild severity. However, there is an advantage in that we can identify the characteristics of help-seeking individuals since the study targeted people with a high interest in mental health services. Third, in this study, we did not gather information on psychiatric history or treatment. Therefore, we cannot determine the confirmed psychiatric characteristics of the community population. Finally, the absence of a clinical sample in our study precludes an assessment of diagnostic validity, requiring subsequent research to explore the utility of the Korean version of the SCAARED in clinical populations.

In conclusion, the analysis of the psychometric properties of the SCAARED indicates that this assessment tool demonstrates good reliability and validity for identifying adults with anxiety disorders in Korea. However, to address the limitations of our study, there is a need for evaluations with larger sample sizes and confirmed clinical populations, as well as other populations. Additionally, as the SCARED has not yet been validated in Korea, a validation study among child and adolescent populations is necessary. This would facilitate the comprehensive use of both instruments across various age groups, from childhood to adulthood, in both clinical and research settings.

## Conclusion

The psychometric properties of the SCAARED indicates that this assessment tool demonstrates good reliability and validity for identifying adults with anxiety disorders in South Korea.

### Electronic supplementary material

Below is the link to the electronic supplementary material.

**Additional file 1:** Factor analysis with principal component analysis

**Additional file 2:** Korean version of the Screen for Adult Anxiety Related Disorders (SCAARED)

**Additional file 3:** Back-translation of the Korean version of the Screen for Adult Anxiety Related Disorders (SCAARED)

## Data Availability

The datasets used and/or analyzed during the current study are available from the corresponding author upon reasonable request.

## Abbreviations

AUC: Area under the curve
BAI: Beck’s Anxiety Inventory
CFA: Confirmatory factor analysis
CFI: Comparative fit index
CMIN: Chi-square minimum
CMIN/df: Chi-square minimum to degrees of freedom
DASS: Depression, Anxiety and Stress Scale
DEAP-40FY: Database for Emotion Analysis Using Physiological and Psychological Assessment by 40FY
df: Degrees of freedom
EFA: Exploratory factor analysis
GFI: Goodness of fit index
KMO: Kaiser-Meyer-Olkin
PAF: Principal axis factoring
PCA: Principal component analysis
RMSEA: Root mean square error of approximation
ROC: Receiver operating characteristic
SCAARED: Screen for Adult Anxiety Related Disorders
SCARED: Screen for Children Anxiety Related Emotional Disorders
SD: Standard Deviation
