# Supplementary material for: Psychometrics of the Korean Version of the screen for adult anxiety related disorders (SCAARED)
Source: BMC Psychiatry. 2024 May 30;24:383. doi: 10.1186/s12888-024-05800-5 (PMC11137947; doi:10.1186/s12888-024-05800-5)
Supplement: Supplementary file 1 — Additional file 1: Factor analysis with principal component analysis [file 12888_2024_5800_MOESM1_ESM.pdf]

Factor analysis with principal component analysis

The factor structures derived from the principal component analysis (PCA) and the principal-axis factoring (PAF) analysis in our study were mostly consistent, with the exception of four items (items 12, 18, 28, and 41) being allocated to different factors, as detailed in Supplementary Table 1.

Supplementary Table 1. Factor analysis for the four-factor solution of the Korean version of the SCAARED (PCA method)

Item	Factor I<br>Generalized anxiety	Factor II<br>Somatic/Panic/agoraphobia	Factor III<br>Social anxiety	Factor IV<br>Separation anxiety
08. It is hard for me to stop worrying	0.788			
23. I am a worrier	0.750			
21. I worry about things working out for me	0.730			
39. I worry about things that have already happened	0.698			
31. When I worry a lot, I feel restless	0.684			
37. I worry about how well I do things	0.670	0.333		
07. I am nervous	0.664			
29. People tell me that I worry too much	0.642			
14. I worry about being as good as other people	0.626			
35. I worry about what is going to happen in the future	0.618			
09. People tell me that I look nervous	0.585		0.321	
05. I worry about people liking me	0.575		0.350	
24. When I worry a lot, I have trouble sleeping	0.538			
44. When I worry a lot, I feel irritable.	0.499	0.363		
12. When I get anxious, I feel like I'm going crazy	0.485	0.429		
28. When I get anxious, I feel like I'm choking	0.455	0.454		
41. I feel nervous when I am with other people and I have to	0.428		0.418	

do something while they watch me (for example: speak, play a sport)				
40. When I get anxious, I feel dizzy		0.676		
06. When I get anxious, I feel like passing out		0.652		
11. I get stomachaches at school, at work, or in public places		0.605		
15. When I get anxious, I feel like things are not real	0.314	0.568		
02. I get headaches when I am at school, at work or in public places		0.547		
01. When I feel nervous, It is hard for me to breathe	0.363	0.547		
36. When I get anxious, I feel like throwing up	0.306	0.515		
25. I get really frightened for no reason at all	0.369	0.514		
17. I worry about going to work or school, or to public places		0.459	0.339	
32. I am afraid of having anxiety (or panic) attacks		0.446		
22. When I get anxious, I sweat a lot		0.440	0.356	
18. When I get anxious, my heart beats fast		0.392		0.365
42. I feel nervous when I go to parties, dances, or any place where there will be people that I don’t know well			0.786	
27. It is hard for me to talk with people I don’t know well			0.764	
10. I feel nervous with people I don’t know well	0.324		0.717	

34. I feel shy with people I don’t know well			0.690	
03. I don’t like to be with people I don’t know well			0.650	
43. I am shy			0.588	
38. I am afraid to go outside or to crowded places by myself		0.417	0.483	
33. I worry that something bad might happen to my family	0.329			0.617
16. I have nightmares about something bad happening to my family				0.613
13. I worry about sleeping alone				0.609
30. I don’t like to be away from my family				0.590
04. I get nervous if I sleep away from home		0.328	0.316	0.518
26. I am afraid to be alone in the house		0.356		0.485
20. I have nightmares about something bad happening to me	0.376			0.391
19. I get shaky				0.327
Eigenvalue	13.6	2.91	2.45	2.07
% of variance	30.9	6.60	6.67	4.70

PCA, principal component analysis; SCAARED, The Screen for Adult Anxiety Related Disorders; All loading greater than 0.30 are reported.

Factor I (Generalized anxiety): Items 5, 7, 8, 9, 12, 14, 21, 23, 24, 28, 29, 31, 35, 37, 39, 41, 44

Factor II (Somatic/Panic/Agoraphobia): Items 1, 2, 6, 11, 15, 17, 18, 22, 25, 32, 36, 40

Factor III (Social anxiety): Items 3, 10, 27, 34, 38, 42, 43

Factor IV(Separation anxiety): Items 4, 13, 16, 19, 20, 26, 30, 33
