# Supplementary material for: Psychometrics of the Korean Version of the screen for adult anxiety related disorders (SCAARED)
Source: BMC Psychiatry. 2024 May 30;24:383. doi: 10.1186/s12888-024-05800-5 (PMC11137947; doi:10.1186/s12888-024-05800-5)
Supplement: Supplementary file 2 — Additional file 2: Korean version of the Screen for Adult Anxiety Related Disorders (SCAARED) [file 12888_2024_5800_MOESM2_ESM.pdf]

Korean version of the Screen for Adult Anxiety Related Disorders (SCAARED)

This is the final Korean version of the Screen for Adult Anxiety Related Disorders (SCAARED). The SCAARED was translated into Korean by an experienced psychiatrist and a clinical psychologist, and back-translated by a bilingual individual, and modifications were made. The final version was reviewed by the original translators.

성인을 위한 불안장애 선별 검사

환자 작성 파트

이름:______________________________ 날짜: ______________________________

아래 문항은 사람들이 어떻게 느끼는지 기술한 것입니다. 각 문장을 읽으신 다음에 “아니다 혹은 거의 아니다”, “다소 그렇다 혹은 때때로 그렇다”, 또는 “매우 그렇다 혹은 자주 그렇다” 중 ***지난 3개월 동안*** 당신을 가장 잘 나타낸다고 생각하는 상태에 표시하십시오.

	0<br>아니다 혹은 거의 아니다	1<br>다소 그렇다 혹은 때때로 그렇다	2<br>매우 그렇다 혹은 자주 그렇다	
1. 긴장할 때, 숨쉬기가 힘들다.				PA/SO
2. 학교, 직장 혹은 공공장소에 있을 때 두통이 생긴다.				PA/SO
3. 잘 모르는 사람들과 함께 있고 싶지 않다.				SOC
4. 집을 떠나 자야 할 때 불안해진다.				SEP
5. 사람들이 나를 좋아할 지 걱정한다.				GA
6. 불안할 때, 쓰러질 것 같은 느낌이 든다.				PA/SO
7. 나는 긴장을 하고 있다.				GA
8. 걱정을 멈추기가 어렵다.				GA
9. 사람들은 내가 긴장하는 것 같다고 말한다.				PA/SO
10. 잘 모르는 사람들과 있으면 긴장한다.				SOC
11. 학교, 직장 혹은 공공장소에 있을 때 복통이 생긴다.				PA/SO
12. 불안할 때, 미칠 것 같다는 느낌이 든다.				PA/SO
13. 혼자 자는게 두렵다				SEP
14. 내가 다른 사람들만큼 잘 할 수 있는지 걱정한다.				GA
15. 불안할 때, 비현실감이 든다.				PA/SO
16. 가족들에게 뭔가 나쁜 일이 일어나는 악몽을 꾼다.				SEP
17. 학교, 직장, 혹은 공공 장소에 가는 것을 걱정한다.				PA/SO
18. 불안할 때, 심장이 빠르게 뛴다.				PA/SO
19. 쉽게 떤다.				PA/SO
20. 나에게 뭔가 나쁜 일이 일어나는 악몽을 꾼다.				SEP

성인을 위한 불안장애 선별 검사

환자 작성 파트

	0<br>아니다<br>혹은<br>거의<br>아니다	1<br>다소 그렇다<br>혹은<br>때때로<br>그렇다	2<br>매우 그렇다<br>혹은<br>자주<br>그렇다	
21. 일이 과연 잘 될까 걱정한다.				GA
22. 불안할 때, 땀이 많이 난다.				PA/SO
23. 나는 프로걱정러이다 (걱정이 많은 사람이다).				GA
24. 걱정이 많아지면 잠을 잘 못 잔다.				GA
25. 아무 이유 없이 두려움을 느낀다.				PA/SO
26. 집에 혼자 있는 것이 두렵다.				SEP
27. 잘 모르는 사람들과 대화하는 게 어렵다.				SOC
28. 긴장하면 숨이 막히는 느낌이 든다.				PA/SO
29. 사람들은 내가 걱정이 너무 많다고 한다.				GA
30. 가족과 떨어지는 게 싫다.				SEP
31. 걱정이 많을 때, 안절부절못한다고 느낀다.				GA
32. 불안 (혹은 공황) 발작이 올까 두렵다.				PA/SO
33. 가족에게 뭔가 나쁜 일이 생기지 않을까 걱정한다.				SEP
34. 잘 모르는 사람들과 있으면 수줍어한다.				SOC
35. 미래에 일어날 일에 대해 걱정한다.				GA
36. 불안할 때 토할 것 같은 느낌이 든다.				PA/SO
37. 내가 얼마나 잘 하고 있는지 걱정한다.				GA
38. 밖이나 사람이 많은 장소에 혼자 나가는 것을 두려워한다.				PA/SO
39. 이미 벌어진 일에 대해 걱정한다.				GA
40. 불안할 때 어지러움을 느낀다.				PA/SO
41. 사람들과 함께 있는데, 그들이 보는 앞에서 무언가를 해야 한다면 (예: 말하기, 운동) 긴장한다.				SOC
42. 파티, 클럽, 혹은 잘 모르는 사람들이 있는 장소에 갈 때 긴장한다.				SOC
43. 나는 수줍음을 탄다.				SOC
44. 걱정이 많아지면, 짜증이 난다.				GA

성인을 위한 불안장애 선별 검사

임상가 작성 파트

이름:______________________________ 날짜: ______________________________

평가:

총점 **23점** 이상이면 **불안 장애** 가능성을 의미함. TOTAL = [ ]

합계 **5점** 이상 (항목 1, 2, 6, 9, 11, 12, 15, 17, 18, 19, 22, 25, 28, 32, 36, 38, 40)은 **공황장애** 혹은 **상당한 신체화 증상** 가능성을 의미함. PA/SO= [ ]

합계 **12점** 이상 (항목 5, 7, 8, 14, 21, 23, 24, 29, 31, 35, 37, 39, 44)은 **범불안장애** 가능성을 의미함. GA = [ ]

합계 **3점** 이상 (항목 4, 13, 16, 20, 26, 30, 33)은 **분리불안장애** 가능성을 의미함. SEP = [ ]

합계 **7점** 이상 (항목 3, 10, 27, 34, 41, 42, 43)은 **사회불안장애** 가능성을 의미함. SOC = [ ]
