# Supplementary material for: Psychometrics of the Korean Version of the screen for adult anxiety related disorders (SCAARED)
Source: BMC Psychiatry. 2024 May 30;24:383. doi: 10.1186/s12888-024-05800-5 (PMC11137947; doi:10.1186/s12888-024-05800-5)
Supplement: Supplementary file 3 — Additional file 3: Back-translation of the Korean version of the Screen for Adult Anxiety Related Disorders (SCAARED) [file 12888_2024_5800_MOESM3_ESM.pdf]

Back-translation of the Korean version of the Screen for Adult Anxiety Related Disorders (SCAARED)

This is the back-translation of the final Korean version of the Screen for Adult Anxiety Related Disorders (SCAARED). The SCAARED was translated into Korean by an experienced psychiatrist and a clinical psychologist, and back-translated by a bilingual individual, and modifications were made. The final version was reviewed by the original translators.

Back-translation of the Korean version of the Screen for Adult Anxiety Related Disorders (SCAARED)

Name:____ Date: _________________________________

The following is a list of statements that describe how people can feel. Read each statement and select an answer that matches how you feel from "Never or almost never", "Somewhat or from time to time", or "Always or very often". Then, report the number that best represents how you felt for the last 3 months.

	**0**<br>**Never or almost never**	**1**<br>**Somewhat or from time to time**	**2**<br>**Always or very often**	
1. When I get anxious, it's hard for me to breathe.				**PA/SO**
2. I get a headache when I'm in school, work or public places				**PA/SO**
3. I don't want to be with people I don't know very well.				**SOC**
4. I get anxious if I have to spend the night away from home.				**SEP**
5. I worry that other people don't like me.				**GA**
6. When I'm anxious, I feel like I am going to faint.				**PA/SO**
7. I feel nervous.				**GA**
8. It's hard for me to stop worrying.				**GA**
9. People tell me that I look nervous.				**PA/SO**
10. I get nervous when I'm with people I don't know very well.				**SOC**
11. I get a stomachache when I'm in school, work or public places				**PA/SO**
12. When I get anxious, I feel like I'm going to lose it.				**PA/SO**
13. I'm afraid of sleeping alone.				**SEP**
14. I worry whether I can do things as well as other people.				**GA**
15. When I'm anxious, things don't feel realistic.				**PA/SO**
16. I have nightmares about something bad happening to my family.				**SEP**
17. I worry about going to school, work or public places				**PA/SO**
18. When I'm anxious, my heart beats faster.				**PA/SO**
19. I get nervous easily.				**PA/SO**
20. I have nightmares about something bad happening to me.				**SEP**

Back-translation of the Korean version of the Screen for Adult Anxiety Related Disorders (SCAARED)

	0<br>Never<br>or<br>almost never	1<br>Somewhat<br>or<br>from time to time	2<br>Always<br>or<br>very often	
21. I worry whether things are going to go well.				GA
22. I sweat a lot when I'm anxious.				PA/SO
23. I am a worrywart (person who worries a lot).				GA
24. When my worries grow, they keep me up at night.				GA
25. I feel scared for no reason.				PA/SO
26. I'm scared of being alone at home.				SEP
27. I find it hard to talk to people I don't know very well.				SOC
28. When I get nervous, I feel like I can't breathe.				PA/SO
29. People tell me that I worry too much.				GA
30. I don't like being apart from my family.				SEP
31. When I get worried, I feel antsy.				GA
32. I'm scared of having anxiety (or panic) attacks.				PA/SO
33. I'm worried that something bad will happen to my family.				SEP
34. I become shy when I am with people I don't know very well.				SOC
35. I worry about things that are going to happen in the future.				GA
36. When I'm anxious, I feel like I'm going to puke.				PA/SO
37. I worry about whether I'm doing a good job.				GA
38. I'm scared of going outside or to a crowded place alone.				PA/SO
39. I worry about what has already happened.				GA
40. When I'm anxious, I feel dizzy.				PA/SO
41. I get nervous when I do something in front of other people (like speaking or exercising).				SOC
42. I get nervous when I go to parties, clubs or places with many people I don't know.				SOC
43. I'm shy.				SOC
44. When I get very worried, I become irritated.				GA
